# Role of artificial intelligence in smart grid – a mini review

**DOI:** 10.3389/frai.2025.1551661

**Published:** 2025-02-04

**Authors:** M. Balamurugan, Kamala Narayanan, N. Raghu, G. B. Arjun Kumar, V. N. Trupti

**Affiliations:** ^1^Department of EEE, Dayananda Sagar College of Engineering, Bangalore, India; ^2^Department of ECE, University of Nevada, Las Vegas, Las Vegas, NV, United States; ^3^Department of EEE, Faculty of Engineering and Technology, JAIN (Deemed-to-be University), Bangalore, India

**Keywords:** smart grid, demand management, machine learning, deep learning, artificial intelligence

## Abstract

A smart grid is a structure that regulates, operates, and utilizes energy sources that are incorporated into the smart grid using smart communications techniques and computerized techniques. The running and maintenance of Smart Grids now depend on artificial intelligence methods quite extensively. Artificial intelligence is enabling more dependable, efficient, and sustainable energy systems from improving load forecasting accuracy to optimizing power distribution and guaranteeing issue identification. An intelligent smart grid will be created by substituting artificial intelligence for manual tasks and achieving high efficiency, dependability, and affordability across the energy supply chain from production to consumption. Collection of a large diversity of data is vital to make effective decisions. Artificial intelligence application operates by processing abundant data samples, advanced computing, and strong communication collaboration. The development of appropriate infrastructure resources, including big data, cloud computing, and other collaboration platforms, must be enhanced for this type of operation. In this paper, an attempt has been made to summarize the artificial intelligence techniques used in various aspects of smart grid system.

## Introduction

The cumulative demand for electricity has directed to a widespread deployment of high-capacity and long-transmission power networks in recent decades (Dranka and Ferreira, 2020). However, difficulties arise from fluctuating and inconsistent energy distribution and the continuous pursuit of enhancing the standards. Incorrect behavior of particular failures can quickly directed to accidents and severe chain reactions, ultimately resulting in a significant outage. The world of today needs a reliable, secure, scalable, interoperable, and economically feasible power system (Bari et al., 2014; Khan et al., 2022). In the future, electrical power systems will be a “smart grid” (Fan et al., 2021), capable of self-healing and providing high-quality, energy-efficient, and reliable electricity.

A smart grid can strengthen the present electrical system by using sustainable sources of energy, which include solar, wind, etc. (Feng et al., 2021; Iris and Lam, 2021). Power transmission and distribution grids need to be upgraded to enhance reliability, economy, efficiency, and security. Need to upgrade the power distribution and transmission grids to improve reliability, economy, efficiency, and security. Introducing excellent communication and control technologies optimizes transmission and distribution grid efficiency. Smart metering, quicker problem analysis, and enhanced network process and management are all altering conventional grid (Shi et al., 2021; Alarifi et al., 2021).

Power supply was compelled to stimulate these methodologies in all of their consequences due to the over-reliance on technology, which increased the need for asset management and tripled productivity. Conventional energy sources are very expensive than smart grid technology. As a consequence, prices for energy have decreased with the advancement of smart grid technology (Iris and Lam, 2021; Kapitonov and Patapas, 2021; Moretti et al., 2017; Panda and Das, 2021). The evolution of traditional grid into smart grid will introduce job opportunities for qualified and unqualified people, in accumulation to supporting advanced technology solutions and financial growth (Acakpovi et al., 2019; Cheena et al., 2020; Matisoff et al., 2020). According to (Sareen et al., 2024), a smart grid reduces power interruptions, improves efficiency, and provides users with more control over distribution of energy and usage (Adamec et al., 2011; El-Hawary, 2016). Customers are more satisfied due to the reduced reliance on conventional grids and the resulting savings (Gungor et al., 2011; Hossain et al., 2016; Wang and Lu, 2013).

**Figure 1:**
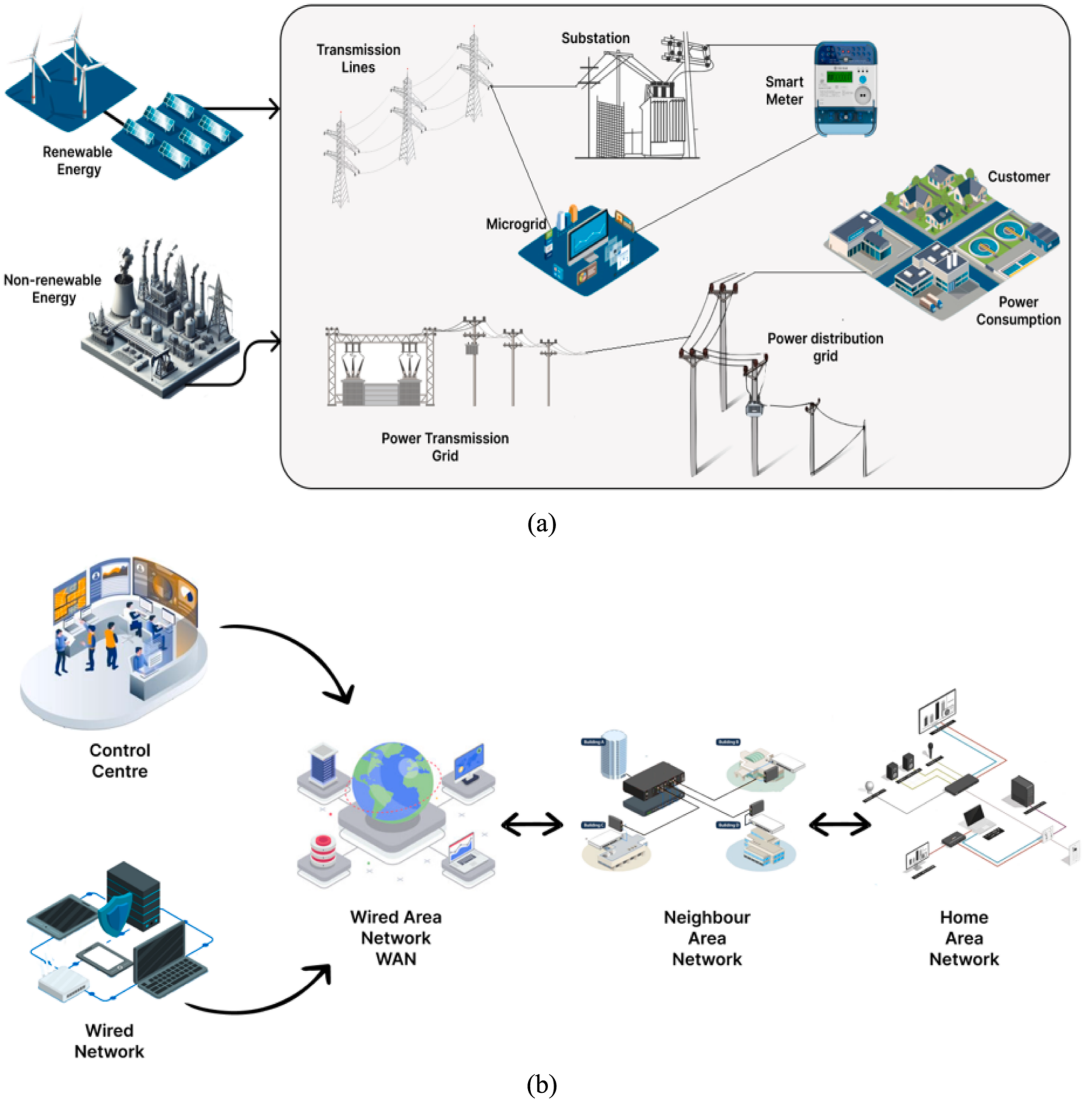
Structure of smart grid. (a) Electrical Network of Smart Grid. (b) Communication Network of Smart Grid.

Figure 1 explains the structure of smart grids. Smart grids are classified as transmission and distribution systems based on their roles. The components of an electrical transmission system are arranged in a circular network with several electrical power generators. A single component breakdown does not significantly affect the transmission network’s performance. The distribution system utilizes radial topology to offer electricity directly to consumers. Minor distribution system failures can severely impact QoS for individual costumers. A distribution system’s self-healing feature isolates defective components from grid and allows for automatic return to regular functioning (Gungor et al., 2013; Wang et al., 2011). A self-healing grid can eliminate power supply disruptions, shorten refurbishment time, and increase renewal load to ensure availability and stability of system. Several international standards have been suggested to provide suitable recommendations for load shedding (IEEE C37.117–2007), condition monitoring analysis (IEC TR 61850–90), connectivity structure (NIST release 3.0), and isolating fault (IEEE C37.114–2004) in context of implementation of smart grid restoration. Smart grids are incorporating emerging technologies, including sensor expertise, microgrid frameworks, energy storage systems, and distributed renewable generation, as well as associated management approaches (Kabalci, 2016; Salkuti, 2020). An energy management system (EMS) in a smart grid (SG) is a computer system that helps balance energy supply and demand while keeping electrical system operations safe, reliable, and cost-effective. EMSs use software and hardware to collect, analyze, and visualize data in real time to control energy flows. The incorporation of Artificial Intelligence (AI) techniques in smart grids has revolutionized the way electricity is produced, transmitted, and distributed. With growing demand for competent and reliable energy supply (Allal et al., 2024), AI has become a crucial component in optimizing grid operations, improving energy efficiency, and enhancing customer experience.

## Artificial intelligence in smart grid

The use of AI in smart grids also enables the incorporation of non-conventional energy sources, such as wind and solar power, into the grid. AI algorithms can analyze data from weather forecasts and energy demand to optimize the output of non-conventional energy sources, reducing the dependence on fossil fuels and improving overall sustainability (Biswal et al., 2024). For instance, a utility company can use AI to analyze data from weather forecasts to predict when solar panels will produce the most energy, and adjust energy production accordingly (Balamurugan et al., 2017). Furthermore, AI can also be used to maximize the use of energy storage devices, such as batteries, to store extra energy produced by non-conventional sources and release it when demand is high. This not only reduces the stress on grid during peak hours and also helps to stabilize the grid and prevent power outages (Balamurugan and Sahoo, 2017).

The benefits of AI in smart grids are numerous, including improved efficiency, reliability, and sustainability. AI can support utilities to lessen energy waste, improve energy efficiency, and enhance customer experience. Additionally, AI can assist to decrease the risk of power outages and brownouts, improving overall grid reliability (Lamnatou et al., 2024). However, the implementation of AI in smart grids also comes with challenges and limitations. One of the significant issues is the readiness and value of data, which is important for training and validating AI terminologies. Utilities must ensure that they have access to high-quality and relevant data, and that they have the necessary infrastructure and resources to progress and examine large quantity of data. Summary of AI techniques in smart grid along with its benefits, challenges and applications is provided in Table 1.

**Table 1:** Artificial intelligence (AI) techniques in smart grid.

AI technique	Problem addressed	Benefits	Challenges	Applications
Machine learning (ML)	Analyses data to forecast energy demand and enhance energy supply	Improves energy efficiency, reduces energy waste, enhances customer experience	Requires high-quality data, can be complex to process.	Load forecasting, demand response, energy storage optimization
Deep learning (DL)	Analyzes complex data sets to detect anomalies and predict energy demand	Improves grid reliability, reduces power outages, enhances customer experience	Requires large amounts of data, can be computationally intensive	Fault detection, predictive maintenance, energy demand forecasting
Natural language processing (NLP)	Analyzes customer feedback and complaints to improve customer experience	Enhances customer experience, improves customer engagement	Requires high-quality data, can be challenging to implement	Customer service chatbots, sentiment analysis
Computer vision	Analyzes images to detect signs of wear and tear on grid infrastructure	Improves grid reliability, reduces power outages, enhances customer experience	Requires high-quality images, can be challenging to implement	Transmission line inspection, equipment monitoring
Reinforcement learning	Optimizes energy storage and release to stabilize the grid	Improves grid stability, reduces energy waste, enhances customer experience	Requires complex algorithms, can be challenging to implement	Energy storage optimization, grid stability management
Transfer learning	Applies pre-trained models to new data sets to improve energy demand forecasting	Improves energy demand forecasting, reduces energy waste, enhances customer experience	Requires high-quality data, challenge to implement	Energy demand forecasting, load forecasting
Clustering	Groups similar data points to identify patterns and drifts in energy dissipation to consumers.	Improves energy efficiency, reduces energy waste, enhances customer experience	Requires high-quality data, can be challenging to implement	Customer segmentation, energy consumption analysis
Decision trees	Analyzes data to make decisions about energy supply and demand	Improves energy efficiency, reduces energy waste, enhances customer experience	Requires high-quality data, can be challenging to implement	Energy supply and demand management, grid optimization

Another challenge is the need for standardization and interoperability, as AI systems must be able to communicate and incorporate with existing grid infrastructure and systems. Utilities must work together to develop industry standards and guidelines for AI implementation in smart grids, and ensure that AI systems are designed to be compatible with existing systems. Despite these challenges, the use of AI in smart grids is becoming progressively widespread, and is anticipated to play a critical role in the energy production and distribution (Kumar and Shivashankar, 2022). As the demand for effective and consistent energy supply continues to grow, AI will become an essential component in optimizing grid operations, improving energy efficiency, and enhancing customer experience.

## AI application in smart grid

The integration of AI and big data analytics in enhancing discretion and safety for demand response modeling within smart grids. Their research emphasizes the critical part of machine learning in managing large-scale information for dynamic pricing and load forecasting while addressing potential cybersecurity threats. The study explores various machine learning frameworks’ efficacy in mitigating risks associated with cyber-physical systems in smart grids, outlining future directions in developing secure, efficient, and resilient energy systems (Reka et al., 2024). The transformative impact of AI in sustainable energy segment, detailing its pivotal roles to enhance operative efficiency, grid optimization, and incorporation of non-conventional energy sources. The study emphasizes AI’s capabilities in managing big data, protect from cyberattacks, and also improves energy management. It outlines three core AI applications: hydrogen and solar power production, management of demand and supply, and AI technology advancement, advocating for stronger regulatory frameworks to harness AI’s full potential in a rapidly evolving energy market (Ahmad et al., 2021).

The deployment of ML and DL within the framework of Industry 4.0, focusing particularly on smart manufacturing and smart grid applications. Their study presents a new architecture for Industrial AI (IAI) and explores the usage of ML and DL in optimizing operations, enhancing cybersecurity, and managing big data challenges in the smart grid sector (Kotsiopoulos et al., 2021). The use of ML and computer vision to determine the angular velocity of wind turbine blades in smart grids. This method not only offers precise localization of turbine hubs and blades but also aids in predicting energy output, which is crucial for planning and optimizing new wind energy facilities (Bahaghighat et al., 2021). A comprehensive review of ML applications across key sustainability sectors including no-conventional energy, smart grids, catalysis, and storage. Their study underscores the predominant use of artificial neural networks due to their superior generalization capabilities and forecasts a rise in ML demand within the energy sector, driven by advances in academia and technology. The review highlights the importance of data generation, management, and security, anticipating that unsupervised and reinforcement learning will play increasingly central roles. Future ML technologies are expected to enable real-time, multi-task processing and integration of various energy systems, enhancing efficiency and promoting sustainable energy management practices. Additionally, the authors point to emerging concerns over data security and ethical standards in the deployment of AI technologies in sustainable development (Rangel-Martinez et al., 2021).

The DL applications in smart grid methodology, highlighting current improvements and prospects. They explore the integration of DL in smart grid for enhanced grid reliability and efficient energy management, emphasizing decentralized decision-making and also discusses state-of-the-art DL paradigms like merged learning, edge intelligence, and distributed computing, identifies the main challenges in deploying these technologies. It advocates for interdisciplinary approaches to overcome computational and communication bottlenecks, suggesting a future focus on Explainable DL to enhance transparency in smart grid systems (Massaoudi et al., 2021). Expert systems (ESs) are artificial intelligence-based systems meant to replicate human knowledge in tackling challenging tasks. Expert systems are being used in SGs for fault diagnostics and decision-making. These technologies can track the grid in real-time, spot abnormalities, and pinpoint the underlying causes of failures such grid instability or equipment breakdown. Usually loaded with a knowledge base covering information about the components of the grid and their behavior under various running situations, the ESs in SGs. The system detects possible problems by means of inference rules, so analyzing data. For instance, the expert system can advise corrective action or immediately reroute electricity to stop harm if a given transformer starts to show indicators of overload. Apart from defect diagnostics, expert systems guide grid management decisions. The ES can advise, for instance, best load distribution plans, preventative maintenance schedules, and grid integration of renewable energy sources. Expert systems help grid operators to save their effort by automating these chores and raise the general system dependability. The application of big data analytics and ML in managing no-conventional energy within smart grids. They develop a framework employing five distinct ML methods to forecast grid stability, utilizing data from a distributed smart grid system. The study reveals high predictive accuracies, highlighting the efficacy of methods like penalized linear regression and convolutional neural networks. Despite the relatively small dataset size, the research demonstrates the potential of cloud computing and real-time analytics in enhancing grid management. Future research is encouraged to expand dataset diversity and include more comprehensive non-conventional energy sources and demand profiles across different countries, aiming to further validate and refine the predictive capabilities of big data analytics in smart grid applications (Mostafa et al., 2022). Deep reinforcement learning is the combination of reinforcement learning and neural networks. It is one of the most effective AI techniques to make optimal choices based on the available data. Deep reinforcement learning technique is used in various stages of smart grid like renewable energy generation, voltage regulation, demand response and resilience enhancement.

The potential of Demand Side Storage (DSS) within smart grids employs a Extreme Learning Machine (ELM) and Genetic Algorithm (GA) to optimize dispatch schedules, aiming to increase generation efficiency and extend the operational lifetime of water pump systems. This multi-objective approach minimizes operational costs and switching frequencies, thereby improving pump longevity. The study showcases the effectiveness of the ELM-GA model through real-time simulation on an IEEE 24 bus test system, highlighting its superiority in handling complex, non-linear optimization problems in smart grid management (Nandkeolyar and Ray, 2022). A novel machine learning-based demand-side management (DSM) engine designed for IoT-enabled smart grids, focusing on improving grid security and energy efficiency. Their proposed system leverages ML classifiers within a resilient agent model to spot and control interruptions, optimizing energy use through advanced management of IoT devices within the grid. Study demonstrates through simulation that their DSM engine not only enhances security against cyber threats but also effectively reduces energy consumption in smart grid environments, proving the incorporation of communication technologies and ML in enhancing grid resilience and efficiency (Babar et al., 2020). The incorporation of technologies within Energy Smart Grids (ESG) by proposing a Multi-Objective Particle Swarm Optimization Algorithm with Competitive Mechanism (MOPSO-CM). This algorithm is designed to optimize the alignment’s extensiveness and exactness in ESG’s Ontology Matching Problem (ESG-OMP), a crucial task for monitoring and efficient energy distribution in smart city environments. The study utilizes a hybrid resemblance measure to differentiate heterogeneous ontology entities and employs competitive updating of particles to enhance solution diversity and convergence. Tested across various standard datasets, MOPSO-CM demonstrates effectiveness in resolving heterogeneous ontology matches, outperforming other Particle Swarm Optimization (PSO) methods. However, the approach faces challenges with complex mappings and large-scale ontologies, suggesting future improvements could include pre-processing strategies and efficiency-enhancing measures (Xue and Tsai, 2022). Classification method comes under the category of supervised learning approach. There are many classifications methods, but mainly linear regression and support vector machines are used for smart grid applications. Especially for optimization problems the outcome can be predicted based on the data availability.

An innovative management approach for smart grids using cloud-fog (CF) technology, which enhances performance through lower delay, better safety, and reduced costs. Their research integrates smart grids with a CF environment, utilizing a novel Honeybee Mating Optimization Algorithm for optimal resource allocation across six fog regions, each containing 500 smart homes. This method is compared to Particle Swarm Optimization (PSO), demonstrating superior performance in load balancing through Digital Twin Virtual Machines (DTVMs). The study, conducted within a digital twin framework on the Eclipse platform, highlights the assistances of DTVMs in smart grid systems, such as scalability, visibility, and collaborative efficiency. This CF-driven scheme significantly improves the management of demand and supply in smart grids, offering a realistic and scalable solution for future grid technologies (Massaoudi et al., 2021; Zhang and Sun, 2023).

The vulnerabilities of smart grid cyber-physical systems, specifically addressing prevalent threat of false data injection (FDI) attacks. These attacks pose serious challenges to grid stability, sustainability, and reliability by corrupting data integrity within smart grids. The study presents a mathematical model of FDI attacks, illustrating their potential impacts on grid operations, economics, and societal trust. It categorizes and evaluates various detection algorithms developed to identify and mitigate such attacks. The study identifies current issues and challenges within this domain and suggests directions for future research, emphasizing the need for a robust cybersecurity framework to enhance resilience against FDI threats in smart grids (Habib et al., 2023).

The incorporation of Industry 4.0 technologies like AI, IoT, and ML in fostering environmental sustainability within manufacturing sectors. Their research highlights how digitalization and smart technologies not only enhance operational efficiency but also significantly contribute to sustainable environmental practices. The study identifies 20 applications where Industry 4.0 can positively impact sustainable production, supply chains, and resource consumption, underscoring the pivotal role of advanced technologies in achieving greener manufacturing processes (Javaid et al., 2022).

## Limitations

However, there are also challenges and limitations to consider, such as:

1. Data quality and accessibility: AI requires high-quality and relevant data to make exact forecasts and decisions.
2. Cybersecurity risks: AI systems can be susceptible to cyber threats, which can negotiate the security and reliability of the grid.
3. Regulatory frameworks: Regulatory frameworks may need to be updated to accommodate the usage of AI in smart electric grids.
4. Public acceptance: There may be concerns about the usage of AI in smart electric grids, particularly around data privacy and job displacement.

Overall, the incorporation of AI techniques in smart electric grids has the potential to transform the way electricity has been generated, transmitted, and distributed, improving efficiency, reliability, and sustainability.

## Conclusion

This paper summarizes the role of AI in smart grid in various stages like control algorithm, optimization strategies and demand side management. In each stage, various AI techniques like DL, ML, Natural language processing techniques utilized by researchers to resolve the problems in smart grid. Although evaluating each method’s standard according to specific performance metrics is difficult, the effectiveness of the approach in resolving the issue has been summarized. Based on the discussion, it is anticipated that AI methods will eventually be a useful instrument for resolving complex smart grid issues. These capabilities would be helpful when considering the importance of AI approaches.
